# Book Notices

**Published:** 1868-01

**Authors:** 


### Book Notices.

A Practical Treatise on *Shock* after Surgical Operations and Injuries, with special reference to Shock caused by Railway Accidents. By EDWIN MORRIS, M.D., F.R.C.S., Surgeon to the Spalding Dispensary and Union Infirmary; author of "A Practical Treatise on Neuralgia." Philadelphia: LIPPINCOTT & CO. 1868.

This is a neatly printed and bound little volume of 89 pages. The subject of which it treats is one of practical interest and importance, and the author gives it a fair consideration.

Synopsis of the Course of Lectures on Materia Medica and Pharmacy, delivered in the University of Pennsylvania, with five Lectures on the Modus Operandi of Medicines. By JOSEPH CARSON, M.D. Fourth Edition; Revised. Philadelphia: HENRY C. LEA. 1867.

This is an octavo volume of 272 pages, the greater part of which is occupied with a simple syllabus or outline notes of Dr. Carson's Lectures. It was designed specially for the use of students attending the Lectures of the author, and, except the five lectures on the Modus Operandi of Medicines, it is of very little interest to any other class of readers.

On Diseases of the Lungs and Air-Passages; Their Pathology, Physical Diagnosis, Symptoms and Treatment. By HENRY WILLIAM FULLER, M.D., Cantab. Fellow of the Royal College of Physicians, London; Physician to St. George's Hospital, etc., etc. From the Second and Revised London Edition. Philadelphia: HENRY C. LEA. 1867.

This is a small sized octavo volume of 479 pages, published in good style. The work is divided into two parts; the first presents a plain, practical, and detailed description of the Principles of Physical Diagnosis, and their application to the investigation of Diseases of the Chest. The second is devoted to the Consideration of the Pathology, Diagnosis, Symptoms, and Treatment of Diseases of the Lungs and their Appendages.

The work, as a whole, is one of real value, and will fully repay even the most busy practitioner for the time and money required for its perusal. For the student, it is one of the best text-books on the subject of which it treats. For sale by W. B. KEEN & Co., 148 Lake Street.

---

Mechanical Therapeutics; A Practical Treatise on Surgical Apparatus, Appliances, and Elementary Operations; Embracing Bandaging, Minor Surgery, Orthopraxy, and the Treatment of Fractures and Dislocations. By PHILIP S. WALES, M.D., Surgeon U.S.N. With six hundred and fifty-two illustrations. Philadelphia: HENRY C. LEA. 1867.

This is a full sized octavo volume of 685 pages, substantially bound in leather, and the text well illustrated by cuts. It seems to embrace everything of importance in the line of mechanical surgery, and is a work of great value, both to the student and the practical surgeon. For sale by W. B. KEEN & Co., 148 Lake Street, Chicago.

